# Probabilistic Analysis for Strain-Hardening Behavior of High-Performance Fiber-Reinforced Concrete

**DOI:** 10.3390/ma12152399

**Published:** 2019-07-27

**Authors:** Seung-Won Choi, Jongkwon Choi, Seong-Cheol Lee

**Affiliations:** 1 Department of Civil and Construction, Chosun College of Science & Technology, Gwangju 61453, Korea; 2 Department of Civil, Architectural and Environmental Engineering, University of Texas at Austin, Austin, TX 78712, USA; 3 Department of Civil Engineering, Kyungpook National University, Daegu 41566, Korea

**Keywords:** probability, high-performance fiber-reinforced concrete (HPFRC), strain hardening, crack, fiber transfer length

## Abstract

The practical application of fiber-reinforced concrete (FRC) in structural components has gained growing interest due to structural advantages such as improved tensile strength, distributed load transfer, crack width control, as well as superior durability. To this end, reliable structural assessment techniques and analytical models have been developed, placing emphasis on tension-softening behavior owing to the bond and pull-out mechanisms of fibers at a local crack. However, these models could not be directly applicable to evaluate the multiple cracking mechanisms of high-performance fiber-reinforced concrete (HPFRC), which exhibits strain-hardening behavior. To overcome this challenge, this paper presents a probabilistic analytical technique. This approach has employed the simplified diverse embedment model (SDEM). Then, an HPFRC member was modeled with multiple segments considering the most probable number of cracks. It was assumed that material properties had a normal probability distribution and were randomly assigned to each segment. To have reliable results, 10,000 analyses were performed for each analysis case and validated using experimental test data. Based on the analysis results, the actual strain-hardening tensile behavior of an HPFRC member could be reasonably predicted with the number of segments chosen on the basis of the fiber length.

## 1. Introduction

Concrete is one of the most widely used construction materials, which has high compressive strength but where the tensile strength is as low as 7~11% of the compressive strength [1]. Typically, concrete under tension exhibits brittle failure with initial cracking when there is no reinforcement. In an effort to improve the post-cracking tensile behavior, many researchers have investigated fiber-reinforced concrete that exhibits post-cracking tensile behavior by adding fiber into the concrete mixture, and as a result, increases ductility of concrete after the crack formation [2,3,4,5,6,7,8,9,10]. Lee et al. [2] developed an analysis procedure for steel-fiber-reinforced concrete (SFRC) elements subjected to shear by implementing constitutive models which are derived from the diverse embedment model (DEM). Lee and Hong [3] evaluated shear strength of ultra-high-performance fiber-reinforced concrete (UHFRC) beams without shear reinforcement. Li et al. [4] investigated shear performance of SFRC beams without web reinforcement. Gali and Subramaniam as well as Husain et al. [5,6] evaluated the improved ductile responses of SFRC beams and flat slabs, respectively. The cyclic response of SFRC slender beams was investigated by Chalioris et al. [7]. Jeong et al. [8] investigated the effect of strength and the aspect ratio of steel fiber on the mechanical properties of high-performance SFRC. Falliano et al. [9,10] investigated the compressive and flexural performance of glass-fiber-reinforced foamed concrete, with emphasis on the effect of fiber properties and curing conditions.

**Figure 1:**
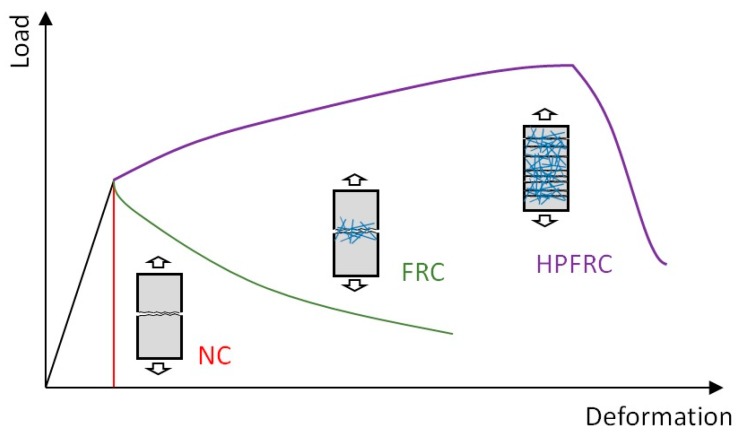
Tensile stress deformation behavior of normal concrete (NC), fiber-reinforced concrete (FRC), and high-performance fiber-reinforced concrete (HPFRC) [11,12,13].

In general, fiber-reinforced concrete (FRC) can be subdivided into two categories based on the post-cracking behavior: The ordinary FRC shows a softening behavior after the first peak load where a single major crack formed within the FRC, whereas high-performance FRC (HPFRC) shows strain-hardening behavior with multiple cracks as the tensile stress attained by fibers is higher than the cracking strength of the concrete matrix (refer to Figure 1) [11,12,13].

Steel fibers, which are the most widely used fibers in FRC, typically provide a bridging effect along the crack interface which allows the ductile behavior of FRC after cracking. To evaluate the post-cracking behavior of an FRC member in tension, many researchers proposed various FRC tension models [14,15,16,17,18]. Marti [14] suggested a stress-crack opening relationship under uniaxial tension considering a random orientation/distribution, and an embedment length of fibers crossing a crack, limiting the maximum crack opening due to fiber length. Voo and Foster [15] proposed the Variable Engagement Model (VEM) which take into account the effective engagement length in the previously suggested model [14]. In this model, the variable embedment fiber length as well as the fiber inclination angle with respect to the loading direction are considered. As such, VEM can predict the tensile behavior of fiber-reinforced concrete well. However, both the models [14,15] are developed on the basis of the frictional bond behavior with a straight fiber. Therefore, those models are more appropriate for the tensile behavior of fiber-reinforced concrete with straight fibers, rather than FRC with end-hooked fibers. To complement this limitation, Lee et al. [16,17] proposed a diverse embedment model (DEM), which can consider both straight and end-hooked fibers. In this model, the frictional bond behavior as well as the anchorage effect were considered in the pullout behavior of a single fiber. With this consideration, the DEM is capable of predicting the tensile behavior of steel-fiber-reinforced concrete with various fiber configurations. Later, Lee et al. [18] proposed a simplified diverse embedment model (SDEM) in which the complicated numerical integration procedure of the DEM was removed, but still maintaining the accuracy on FRC tensile behavior prediction. Cunha et al. [19] presented a numerical approach to simulate the flexural behavior of FRC beams exhibiting the tension-softening behavior.

Many models [14,15,16,17,18] mainly focused on the prediction of tensile behavior of fiber-reinforced concrete exhibiting a tension-softening behavior with a single dominant crack (refer to Figure 1). In these models, the tensile stresses owing to steel fibers are assumed to be proportional to the fiber content. However, Lee et al. [20] showed that the efficiency of steel fibers within concrete decreases as the fiber contents increased. In order to compensate this effect, the VEM employed a simple correction coefficient to consider the decreasing steel fiber efficiency due to the increased fiber content. Even though Lee et al. [20] proposed a simple model for the fiber efficiency, there was no theoretical basis for the derivation of the model. Therefore, a simple and direct employment of the pre-developed models on the basis of the softening behavior may not be applicable to predict the tensile behavior of HPFRC.

To overcome this shortcoming with the existing SFRC tension models, in this study, an analytical method is presented to evaluate tensile behavior of HPFRC which exhibits strain-hardening behavior with multiple cracks due to a high volumetric ratio of fibers. Specifically, in an effort to account for the random multi-cracks formation due to the increased volumetric fiber contents (HPFRC), probabilistic methods were employed to predict the strain-hardening behavior.

## 2. Strain-Hardening Behavior of HPFRC

**Figure 2:**
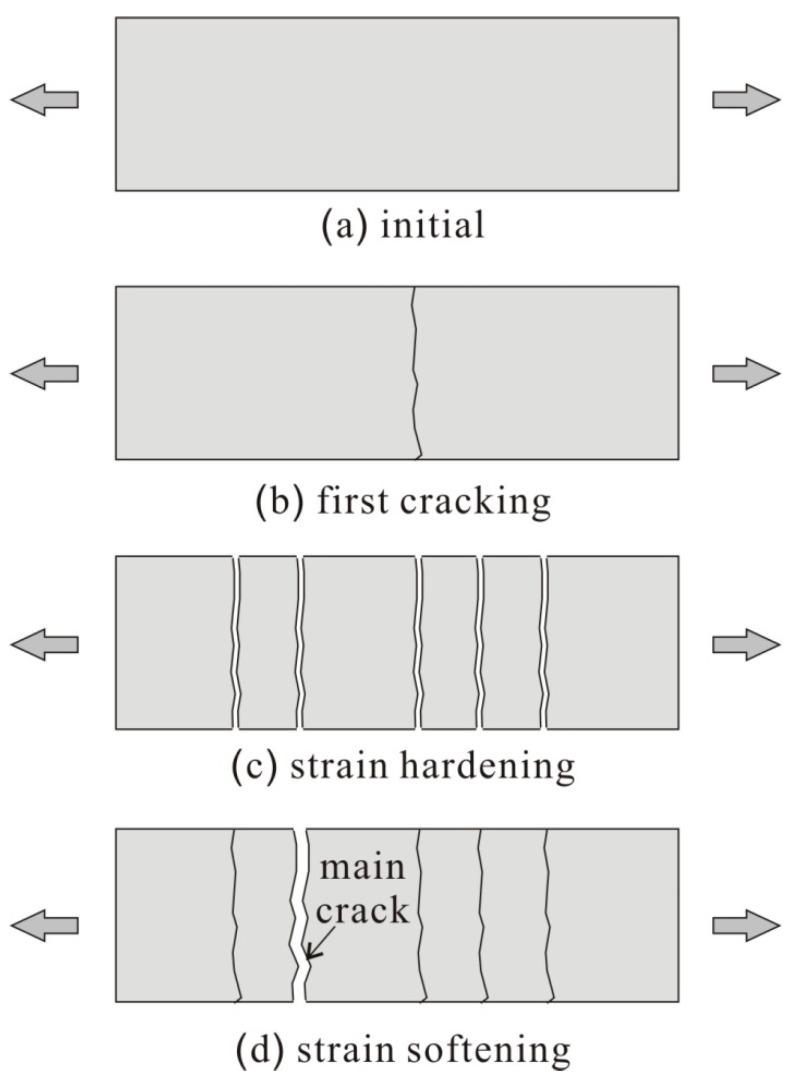
Cracking pattern of HPFRC subjected to tension. (**a**) initial; (**b**) first cracking; (**c**) strain hardening; (**d**) strain softening.

This section presents the sequential multi-cracking phenomena which is the main mechanism of the strain-hardening behavior of HPFRC under uniaxial tensile loading conditions. For a better understanding of the sequential multi-cracking phenomena, the sequential cracking behavioral model presented by Shin et al. [21] has been employed. Figure 2 shows the sequential cracking mechanisms of HPFRC subjected to uniaxial tensile deformation. As the tensile deformation increased, the tensile stresses within the HPFRC increased, and once the tensile stress exceeds the cracking strength of the concrete matrix, the first crack initiates at the section where the cracking strength of concrete matrix is the lowest (Figure 2b). In ordinary FRC with low or moderate fiber volumetric contents (i.e., not more than 1.0% in general), tensile stress attained by fibers is typically lower than the cracking strength of the concrete matrix so that only a single dominant crack is formed with no additional crack. Therefore, a tension-softening behavior is observed in the ordinary FRC due to a single dominant crack where deformation of the FRC member is localized. However, in case of HPFRC, which has relatively higher volumetric fiber contents (i.e., more than 1.5% in general), tensile stress attained by fibers exceeds the cracking strength of the concrete matrix. As a result, additional cracks are formed along the HPFRC member, which resulted in a multiple-crack pattern as illustrated in Figure 2c. On the other hand, as presented in the DEM [16], tensile stress in the concrete matrix increases as the distance from a crack increases because the tensile stress by fibers is transmitted to the concrete matrix by frictional bond and mechanical pulling-out. Once enough multi-cracks form within the HPFRC member, no additional crack likely initiates. This is because the length of each segment is less than the characteristic transfer length in a HPFRC member, as such tensile stress of the concrete matrix cannot exceed the cracking strength of concrete. During this stage, the tensile deformation of HPFRC increases as cracks opens without additional crack formation. Once the applied tensile stress reaches the maximum tensile stress attained by fibers at the section where the fiber contribution is the lowest, the tensile stress of HPFRC cannot increase. At this stage, the tensile deformation of the HPFRC member is concentrated at the crack with the lowest fiber contribution. After surpassing the maximum tensile stress at the deformation-concentrated crack, the HPFRC member exhibits a post-peak tensile behavior with the localized crack width opening at the crack. On the other hand, the crack width of the other cracks decreases due to the applied tensile stress in HPFRC decreasing, as illustrated in Figure 2d.

**Figure 3:**
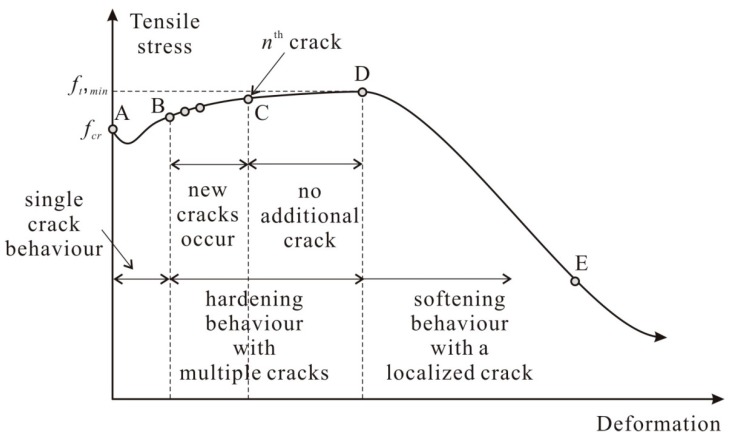
Tensile stress-deformation response in HPFRC.

Figure 3 depicts the typical tensile stress uniaxial deformation response of a HPFRC member considering the multiple crack formation. The first crack forms at the most vulnerable section where the concrete cracking strength is the lowest. Upon the first cracking, instantaneous tensile stress reduction may occur due to softening of the concrete matrix (A to B in Figure 3). As the deformation increased, fibers at the crack engaged, as such the tensile stress gradually increased. Then, as tensile stress at the crack reaches the cracking strength of the concrete matrix at another section, additional cracks begin to occur sequentially (points B through C in Figure 3). If the crack spacing is insufficiently large to cause an additional crack (i.e., the tensile stress transmitted from fibers to the concrete matrix is less than the cracking strength of concrete matrix), no further crack occurs while tensile deformation of HPFRC increases with crack width opening at all cracks, until the maximum tensile stress of HPFRC is reached (C to D in Figure 3). After surpassing the maximum tensile stress attained by fibers at the weakest crack section, HPFRC exhibits softening behavior as the localized crack is dominant (D to E in Figure 3).

**Figure 4:**
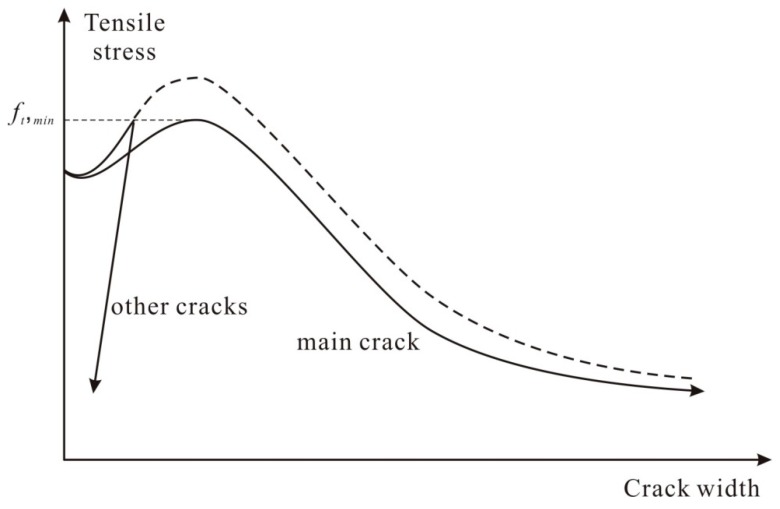
Tensile stress-crack width opening response at the main crack and the others.

Figure 4 shows the conceptual tensile stress-crack width opening responses at the localized crack and the other cracks. As shown in Figure 4, after reaching the maximum tensile stress attained by fibers at the localized crack, the weakest section exhibits the post-peak tension softening behavior while the other cracks follow the unloading path.

## 3. Analytical Model for an HPFRC Element Subjected to Uniaxial Tension

Unlike conventional FRC members that exhibit tension softening behavior with a single dominant crack, HPFRC members exhibit strain-hardening behavior with multiple cracks since tensile stress attained by fibers is typically larger than the cracking strength of the concrete matrix. Therefore, to reasonably predict the tensile behavior of an HPFRC member, an analytical method which is capable of taking into account multiple cracking phenomena (i.e., strain hardening) should be developed. To account for the sequential multiple cracks formation over the strain hardening phase, the probabilistic distribution of material properties can be considered. In addition, the tension softening behavior after the localized crack width opening should be considered in the analytical method. 

In this section, an analytical procedure to predict the tensile behavior of HPFRC considering probabilistic distribution of material properties is presented in an effort to predict both the strain-hardening behavior due to sequential multi-cracking phenomena and the softening behavior after the localized cracking.

### 3.1. A Sequential Cracking Behavior Model with Multi-Segments

**Figure 5:**
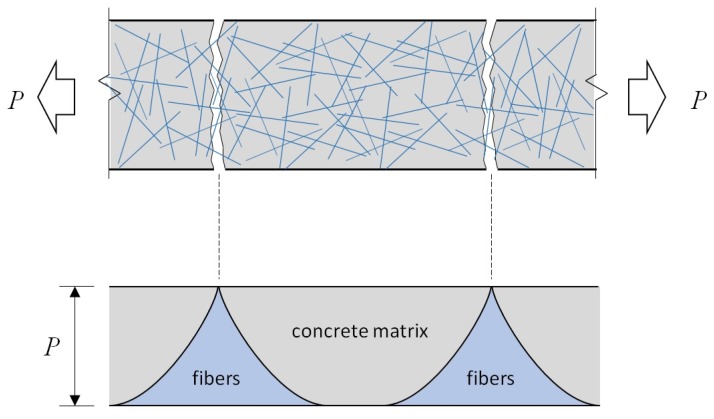
Concrete matrix and steel fiber tensile stress distributions between cracks.

As aforementioned, the first crack is formed when the tensile stress applied to the member exceeds the cracking strength of the concrete matrix. Due to the nonhomogeneous characteristic of concrete, the first crack forms at the location within an HPFRC member, which has the minimum cracking strength of the concrete matrix. Then, fiber contribution on the tensile stress initiates through bridging the crack through frictional bond and mechanical pulling-out behaviors. In the case of HPFRC, the fiber contribution withstands the tensile load applied to the member at the crack interface. Since the cracking strength of concrete matrix has variation along the member, it can be considered that a new crack occurs whenever the tensile strength of the concrete matrix at another section is reached. In this case, based on the DEM [16,17], if the tension softening effect of the concrete matrix is ignored, tensile stress in the concrete matrix is zero at a crack while tensile stress by fibers is at the maximum (refer to Figure 5). Since the tensile stress attained by fibers transmits to the concrete matrix through pull-out behavior of fibers, tensile stress of the concrete matrix increases with a distance from the crack surface. Consequently, it can be inferred that the minimum distance to an immediately adjacent crack can be determined by the effective transfer length of fibers. 

In case of reinforced concrete members under tension, average crack spacing can be assumed to be 1.33 times the transfer length of a rebar in concrete [22], with consideration of local variation in concrete stress due to the bond mechanism between rebar and concrete. In a similar manner, 1.33 times the fiber length can be assumed as the average crack spacing in an HPFRC member, since the fiber length is considered as the transfer length that the tensile stress attained by fibers entirely transmits to the concrete matrix [16,17]. Since the effect of the fiber shape on the local stress variations presented in Figure 5, when the crack width is less than 0.1 mm corresponding to the strain-hardening behavior of an HPFRC member, this assumption can be used regardless of the fiber shape.

**Figure 6:**
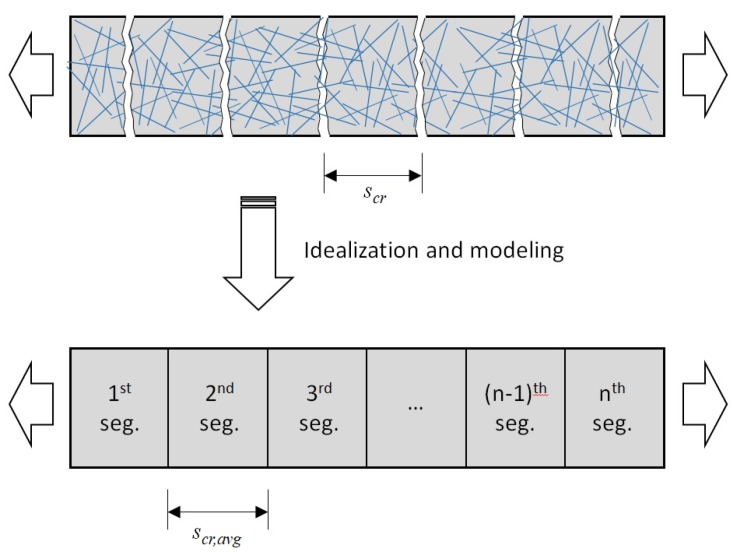
Analytical model with segments for an HPFRC member subjected to uniaxial tension.

Therefore, as shown in Figure 6, to analyze the tensile behavior of HPFRC members, the length of each segment can be assumed to 1.33 times the fiber length. Thus, the maximum anticipated number of both cracks and segments can be simply obtained by dividing the member length by the transfer length of the fiber (i.e., 1.33 times the fiber length). This multi-segment model coupled with the consideration of variation of the cracking strength of the concrete matrix and the tensile stress owing to the steel fibers can be used to predict the HPFRC tensile behavior. To validate the proposed multi-segment model, in this paper, the parametric study was performed using various number of segments. 

### 3.2. Constitutive Models for the Materials

In general, the tensile stress ($f_{t,FRC}$) of FRC consists of the tensile stress attained by fibers ($f_{f}$) and the tensile stress due to the concrete matrix tension softening ($f_{ct}$) as follows [15,16,17,18]:

(1) $$f_{t,FRC}=f_{f}+f_{ct}.$$

The tensile stress attained by fibers can be evaluated for a given crack width, with consideration of the random distribution of fibers and fiber volumetric ratio in a member. To evaluate the tensile stress attained by end-hooked steel fibers, this paper employed the SDEM [18], which showed excellent agreement with test results. In the SDEM, the tensile stress attained by end-hooked steel fibers can be evaluated with consideration of the frictional bond behavior and mechanical anchorage effect separately, as follows:

(2) $$f_{f}=f_{st}+f_{eh}$$

where $f_{st}$ is the tensile stress due to the frictional bond behavior of the straight part of fibers, and $f_{eh}$ is the tensile stress due to the mechanical anchorage effect of the hooked end of fibers.

The tensile stress due to the frictional bond behavior of fibers $f_{st}$ can be obtained using the fiber volumetric ratio ($V_{f}$), the fiber length ($l_{f}$), the fiber diameter ($d_{f}$), and the maximum frictional bond strength of a fiber ($\tau_{f,\max}$), as follows [18]:

(3) $$f_{st}=\alpha_{f}V_{f}K_{st}\tau_{f,\max}\frac{l_{f}}{d_{f}}{\left(1-\frac{2w_{cr}}{l_{f}}\right)}^{2}$$

where $\alpha_{f}$ is the fiber orientation factor which is 0.5 for an infinitely large section and can be greater than 0.5 for a relatively small section [18], $w_{cr}$ is a crack width, and $K_{st}$ is defined as follows:

(4) $$K_{st}=\{\begin{array}{cc} \frac{\beta_{f}}{3}\frac{w_{cr}}{s_{f}} & \mathrm{for}\;w_{cr}\leq s_{f}\\ 1-\sqrt{\frac{s_{f}}{w_{cr}}}+\frac{\beta_{f}}{3}\sqrt{\frac{s_{f}}{w_{cr}}} & \mathrm{for}\;w_{cr}>s_{f} \end{array}$$

where $s_{f}$ is the slip corresponding to the maximum frictional bond strength, and $\beta_{f}$ is the coefficient to consider the effect of a larger embedded length of the fiber, and it has been analytically obtained as 0.67 [18].

In Equation (2), $f_{eh}$ is the tensile stress due to the mechanical anchorage effect by the end-hook of fibers. It can be evaluated with the following equation:

(5) $$f_{eh}=\alpha_{f}V_{f}K_{eh}\tau_{eh,\max}\frac{2\left(l_{i}-2w_{cr}\right)}{d_{f}}$$

where $\tau_{eh,\max}$ is the equivalent bond strength to consider the mechanical anchorage effect, and $l_{i}$ is the distance between mechanical anchorages (i.e., end-hooks) in an end-hooked fiber. $K_{eh}$ is a factor representing average pullout stresses due to mechanical anchorage of end-hooked fibers, and it can be defined as follows [18]:

(6) $$K_{eh}=\{\begin{array}{cc} \beta_{eh}\left[\frac{2}{3}\frac{w_{cr}}{s_{eh}}-\frac{1}{5}{\left(\frac{w_{cr}}{s_{eh}}\right)}^{2}\right] & \mathrm{for}\;w_{cr}\leq s_{eh}\\ 1+\left(\frac{7\beta_{eh}}{15}-1\right)\sqrt{\frac{s_{eh}}{w_{cr}}}-\frac{2{\left(\sqrt{w_{cr}}-\sqrt{s_{eh}}\right)}^{2}}{l_{f}-l_{i}} & \mathrm{for}\;s_{eh}\leq w_{cr}\leq \frac{l_{f}-l_{i}}{2}\\ {\left(\frac{l_{i}-2w_{cr}}{2l_{i}-l_{f}}\right)}^{2}K_{eh,i} & \mathrm{for}\;\frac{l_{f}-l_{i}}{2}\leq w_{cr}<\frac{l_{i}}{2} \end{array}$$

where $s_{eh}$ is a slip corresponding to the maximum tensile force due to the mechanical anchorage of an end-hooked fiber, and $K_{eh,i}$ is $K_{eh}$ at $w_{cr}=\left(l_{f}-l_{i}\right)/2$ and $\beta_{eh}$ is a coefficient to consider the effect of fiber slip on a longer embedded side of a fiber, and it is set to 0.76 in general [18].

The tensile response considering the tension softening effect of the concrete matrix can be evaluated with an exponential function as follows [15]:

(7) $$f_{ct}=f_{cr}e^{-cw_{cr}}$$

where $f_{cr}$ is the cracking strength of the concrete matrix, and c is a coefficient to consider the effect of the material type, which can be taken as 15 for the concrete matrix with coarse aggregate [15].

### 3.3. Probabilistic Distribution on the Material Properties

**Figure 7:**
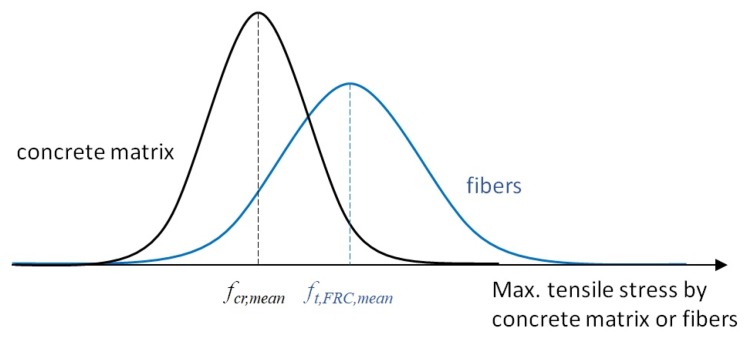
Normal distribution of the tensile stress due to steel fibers.

In an effort to reasonably predict tensile behavior of an HPFRC member, the tensile stress-crack width behavior for each crack should be considered in the analytical procedure to account for sequential multiple cracking phenomena described in the previous sections. More specifically, to account for the uncertainty on the material properties along a UHFRC member, probabilistic variations were assumed for cracking strength of the concrete matrix and the tensile stress due to fibers along the HPFRC member, respectively. To apply the probabilistic variations on the material properties on the analysis, the HPFRC member was modeled with several segments on the basis of the average crack spacing criteria. Then, a stochastic distribution of both cracking strength of the concrete matrix and tensile stress due to fibers was considered for each HPFRC segment. Figure 7 shows normal distributions of both the concrete matrix and fiber with respective material properties. In typical HPFRC, the average cracking strength is lower than the average tensile stress attained by fibers due to strain-hardening behavior. However, the standard deviation of tensile stress attained by fibers is larger than that of concrete cracking strength. This is mainly due to the fiber dispersion and fiber orientation within the concrete matrix [23,24]. Noted that the normal distribution in this case is a little different from a lognormal distribution since the average is not close to zero considering the standard deviation of the material properties.

To determine the occurrence of cracks, the stochastic distribution of the concrete matrix cracking strength was considered to be 10% of the CoV (coefficient of variation) based on the literature [25]. In the uniaxial tension test, the first cracking strength of concrete matrix in an HPFRC member, $f_{cr,test}$, is measured, and it can be considered as the minimum cracking strength of the concrete matrix along the member. Hence, the actual mean value on the cracking strength of the concrete matrix is generally larger than the first cracking strength. Based on a normal distribution, the mean value on the cracking strength of the concrete matrix ($f_{cr,mean}$) can be calculated with consideration of the member length and number of cracks, as the following equation:

(8) $$f_{cr,mean}=\frac{f_{cr,test}}{1-N_{cr}\cdot {\mathrm{CoV}}_{fcr}}$$

where $f_{cr,test}$ is the cracking strength corresponding to the first crack observed through uniaxial tension tests, $N_{cr}$ is a possible number of cracks (i.e., a number of segment in the analysis), and ${\mathrm{CoV}}_{fcr}$ is a CoV for a probabilistic distribution on the cracking strength of the concrete matrix.

In a similar manner, the average tensile stress attained by fibers can be calculated using Equations (2) through (8). The tensile stress attained by fibers is typically assumed to be proportional to the number of fibers bridging a crack since it is directly related to a fiber orientation factor as indicated in Equations (3) and (5). Regarding the number of fibers bridging a crack, Lee et al. [22,23] evaluated the fiber orientation factor by counting the number of fibers crossing a crack surface. They obtained a 20% CoV for the fiber orientation factor regardless of the shape of the cross-section of concrete specimens. Therefore, a 20% CoV was assumed for the probabilistic distribution of the tensile stress attained by fibers.

## 4. Verification of the Proposed Analysis Model

### 4.1. Summary of the Test Program by Lee et al. [20]

**Figure 8:**
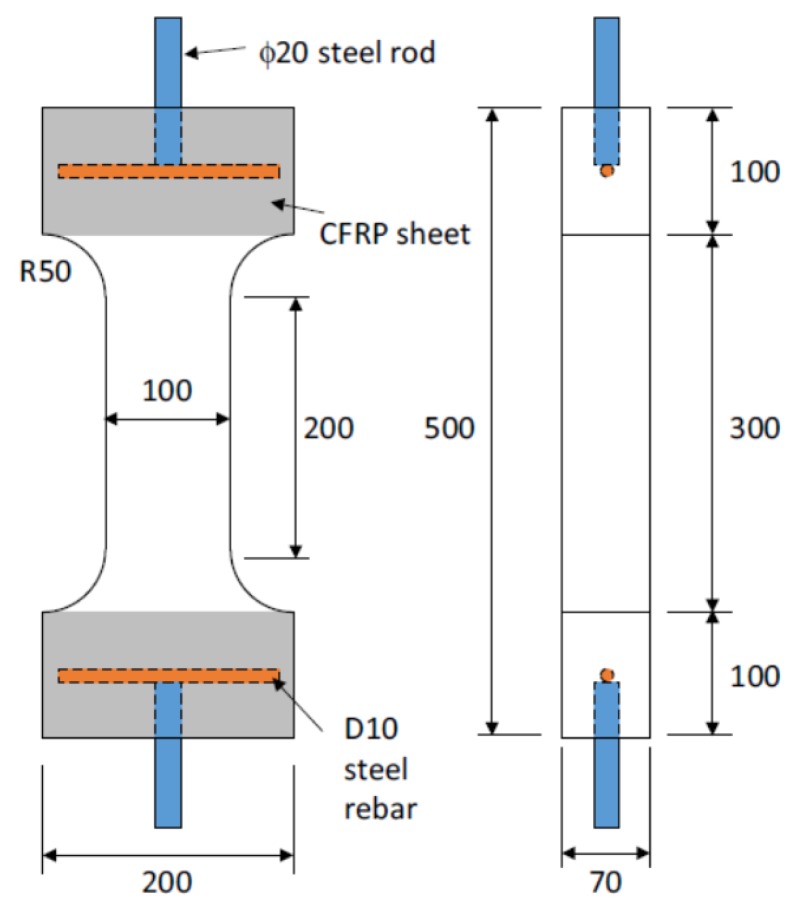
Details of the specimens tested by Lee et al. [20].

Lee et al. [20] conducted uniaxial tension tests on a series of dog-bone shaped specimens. Among those test series, HF3V4 specimens have been chosen since they exhibited a typical strain-hardening behavior with multiple crack formation owning to a 2% fiber volumetric ratio. The overall dimensions of the dog-bone specimen are summarized in Figure 8. Uniaxial tension was applied the specimens at a constant displacement rate of 0.01 mm/s. Due to the geometry of the dog-bone specimen, cracks were induced in the middle of the specimen with the reduced cross-sectional region. In HF3V4 specimens, concrete was mixed with end-hooked fibers which had a length of 30 mm, a diameter of 0.38 mm, and a tensile strength of 2300 MPa. The compressive strength and the first cracking strength of the specimens were 83.0 and 3.40 MPa, respectively. Through the test, it was observed that the average on the number of cracks was 5.5, and the average on the maximum tensile stress was 4.46 MPa. More details can be found in Lee et al. [20].

### 4.2. Analysis Results and Comparison with the Test Results

For the probabilistic analysis of HPFRC subjected to tension, CoVs were assumed to be 10% for cracking strength of the concrete matrix and 20% for tensile stress attained by fibers, respectively, as explained above in the previous section for the probabilistic distribution on the material properties. Then, the probabilistic distribution on the material properties was applied through the segments in the analytical model. It is noted that the given values in the analysis were only two material properties measured through the test; the compressive strength and initial cracking strength of HPFRC. To investigate the effect of the number of segments on the analysis results, the number of segments was also considered as an analysis variable. In addition, to attain reliable evaluation, 10,000 material properties distributions were analyzed per each analysis case corresponding to the number of segments. In this section, the number of expected cracks and the tensile behavior of HPFRC were investigated with a varying total number of segments. Through comparison of the predictions with the test results, the proposed analytical procedure is verified.

#### 4.2.1. Number of Cracks

To identify the optimal number of segments on the analytical model, probabilistic analyses were conducted and the probabilities corresponding to the number of cracks were evaluated. The number of segments chosen for each analysis case were 1, 3, 5, 10, 15, and 20. As aforementioned, the cracking strength of the concrete matrix and the tensile stress attained by fibers were assumed to have a normal distribution with respective CoVs. Then those values were randomly selected and assigned to each segment as material properties.

**Figure 9:**
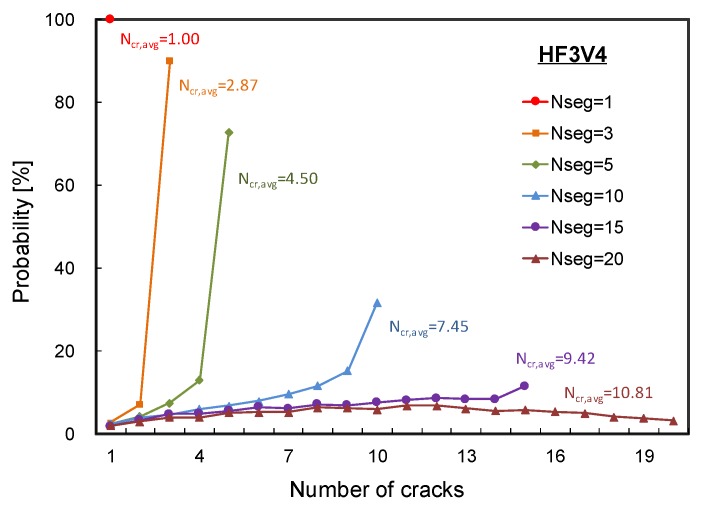
Probabilistic distribution on the predicted number of cracks.

Figure 9 shows the probabilities for the number of cracks predicted by the proposed analytical model. In the figure, each point indicates the probability that the corresponding number of cracks were predicted on the analysis. For example, in the case of the analysis with five segments, the possibility of five cracks was evaluated to be 72.9%. As shown in the figure, the number of cracks predicted by the probabilistic analysis model using normal distributions on the two material properties also shows a probabilistic distribution. It should be noted that the number of average predicted cracks ($N_{cr,\mathrm{avg}}$) for each analysis case was evaluated in an average manner using a set of 10,000 analysis results. In some analysis cases, especially with a large number of segments, some of the segments were uncracked in the analyses. In addition, when the number of segments was smaller than 10, it was highly predicted that most of the segments were cracked on the analysis results. On the other hand, when the number of segments was set to 20, it was highly possible that the number of cracked segments was 11 or less with a probability of 50%. This indicates that an increasing number of segments does not necessarily improve the accuracy on the prediction for the number of cracks. 

As shown in the figure, when the number of segments was less than five, the predicted average number of cracks was close to the number of segments. This means all the segments were cracked in high possibility. On the other hand, when the number of segments was larger than 10, the predicted average number of cracks was considerably smaller than the number of segments. Therefore, considering that an appropriate number of segments in the analysis may depend on the length of an HPFRC member. It can also be inferred that the larger the member length, the more likely it is that the cracks are not well distributed along the long member.

The average number of cracks reported in Lee et al. [20] was 5.5 for HF3V4 specimens. Therefore, it can be inferred that the reasonable number of segments for the specimens is between 5 and 10 when the specimens are analyzed. Notably, the length of the possibly cracked region in the specimen was at least 200 mm, not including the region with a varying cross-section, and up to 300 mm including the region. It is interesting that the appropriate length of a segment in the analysis can be assumed to be 1.33 times the fiber length, since the predicted number of cracks was close to the test result when the number of segments was set to 5 or 10.

#### 4.2.2. Tensile Behavior of HPFRC

**Figure 10:**
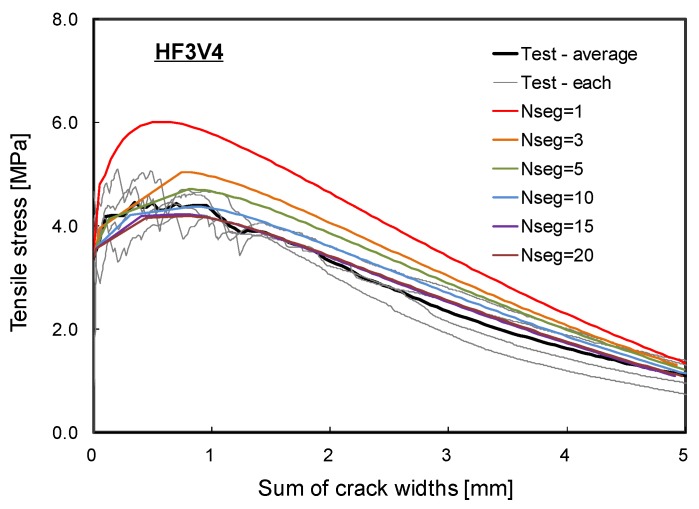
Comparison of the prediction and the test results for the tensile behavior.

To predict the strain-hardening behavior of HPFRC, the proposed probabilistic analyses were conducted using the idealized HPFRC with various number of segments (i.e., $N_{seg}=$ 1, 3, 5, 10, 15, and 20). In Figure 10, the predicted tensile stress total crack width responses obtained from the proposed probabilistic analyses were compared with the experimentally obtained uniaxial tensile behaviors of HF3V4 specimens. It should be noted that the response with a single segment model (i.e., $N_{seg}=1$) is identical to the prediction by the SDEM.

In the analysis case with a single segment, the predicted maximum tensile stress was higher than the experimental test results. It can be seen in Figure 10 that the maximum tensile stress predicted by the proposed analysis decreased as the number of segments in the analysis model increased. This indicates that the analysis model using a single or few segments (i.e., the number of segments is less than the realistic number of cracks) is not representative of the actual tensile behavior of HPFRC: The model is not capable of capturing multiple cracks with sequential cracking patterns; the maximum tensile stress is overestimated; and there are unrealistic pre-peak strain hardening and post-peak softening behaviors (Figure 10).

On the other hand, an increasing number of segments in the analysis model is not always necessarily improving the accuracy of the analysis results. When the HPFRC specimen was modeled with 20 segments (i.e., the number of segments was larger than the realistic number of cracks), the maximum tensile stress predicted by the analysis was slightly lower than the test result. This tendency is obvious because the minimum among the maximum tensile stresses attained by fibers along the cracks decreased due to the natural characteristic of a probabilistic distribution on the material properties. An over-predicted number of cracks or segments caused unnecessary reduction on the tensile stress of HPFRC, which resulted in reduced strain-hardening behavior. 

Therefore, the optimal number of segments should be carefully chosen to reasonably represent the actual cracking behavior of an HPFRC specimen. In Figure 10, the tensile stress total crack width responses of the analysis model using 5 and 10 segments showed good agreement with the experimental test results of HF3V4 specimens. Considering the average number of cracks observed from the test program was 5.5 on the average through 4 specimens, one can conclude that the number of segments should be chosen with the most realistic number of cracks estimated to accurately predict the actual tensile behavior of an HPFRC member. One of the ways to define the optimal number of segments is using a fiber length. As noted in Section 3 of this paper, the length of a segment can be assumed as 1.33 times the fiber length. The fiber length for the experimental program was 30 mm, so the length of a segment could be set to 40 mm. The main test region was the straight portion (i.e., 200 mm in the mid-portion as depicted in Figure 8) of the dog-bone specimen in which most of the cracks were formed during the uniaxial tension test. In some cases, the cracks were also formed at the tapered section, as such the cracking region of the dog-bone specimen can be extended to 300 mm. Therefore, the possible number of cracks can be 5 to 7.5. Since the observed average number of cracks was 5.5, the initial assumption for the length of a segment based on 1.33 times the fiber length is deemed a rational assumption on average crack spacing to estimate the number of cracks/segments. 

In Table 1, the tensile stresses and sums of crack widths predicted by the analyses have been summarized for specific points depicted in Figure 3; ‘A’ corresponding to initiation of the first crack, ‘B’ corresponding to initiation of the second crack, ‘C’ corresponding to occurrence of the last crack, and ‘D’ corresponding to the maximum tensile stress of the HPFRC member. Regardless of the number of segments, the predicted tensile stress corresponding to occurrence of the first crack was quite similar since the cracking strengths of segments were assumed to follow a normal distribution with Equation (8). This indicates that the probabilistic distribution on the material properties was reasonably generated. On the other hand, the maximum tensile stress of the HPFRC was predicted to decrease as the number of segments in the analysis model increased. Considering the probabilistic distribution on the tensile stress attained by fibers, increase on the number of cracks is obviously resulted in decrease on the maximum tensile stress of a HPFRC member since the tensile stress attained by fibers in the weakest cracked section decreases. In addition, under the same tensile stress, the total sum of crack widths increases as the number of segments in the analysis model increases. This can be inferred that all cracks in the member gradually opens before reaching the maximum tensile stress. However, for the tensile stresses or the total sum of crack widths corresponding to the second and subsequent crack initiation, the variation in the prediction of the probabilistic analysis was relatively large. Therefore, it is deemed that the information about the first cracking strength, the maximum tensile stress, and the corresponding deformation can be used to develop a simple model for the tensile behavior of HPFRC; it may not be necessary to focus on the information about the tensile stresses and crack widths corresponding to sequential cracks.

**Figure 11:**
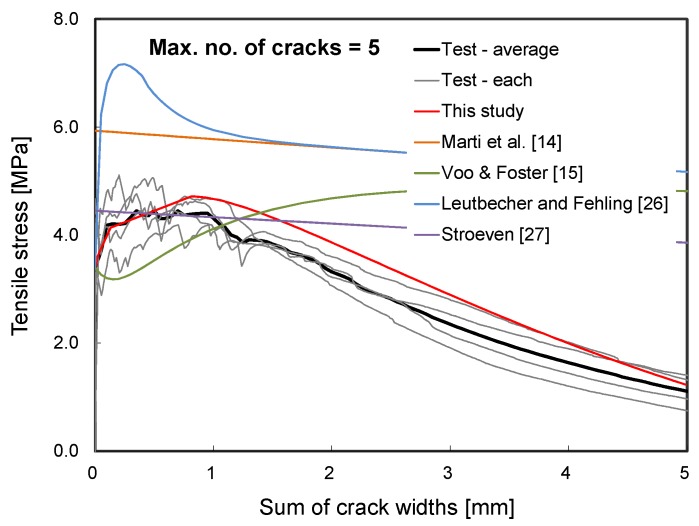
Comparison with FRC tension models.

In Figure 11, analytical predictions with several FRC tension models [14,15,26,27] were also compared with the test results and the predictions by the proposed analytical procedure. Note that five cracks were considered when the FRC tension models were employed to evaluate the tensile stress total crack widths response of the HPFRC member. As can be seen in the figure, conventional FRC models significantly overestimated ductility or the maximum tensile stress since they were designated for the conventional FRC exhibiting the tension-softening behavior. On the other hand, the proposed analysis procedure well captured the test results when an appropriate number of cracks were considered. Therefore, it can be concluded that the proposed analytical procedure can be useful to evaluate the actual tensile behavior of HPFRC members.

## 5. Conclusions

This paper presented a probabilistic analysis model developed on the basis of an SDEM model to predict the strain-hardening behavior of UHFRC. The model takes into account the probabilistic distribution of the material properties (i.e., cracking strength of the concrete matrix and tensile stress attained by fibers). In an effort to consider the uncertain nature of the material properties, a normal distribution was assumed for both cracking strength of the concrete matrix and the maximum tensile stress due to fibers, and typically known CoVs of respective materials were considered to assign the variation following the normal distribution along a member. Owing to the developed probabilistic analytical procedure, the strain-hardening behavior of an HPFRC member could be rationally evaluated with the conventional FRC tension model originally designated for the tension-softening behavior. Through the theoretical approach and verifications conducted in this paper, several conclusions were drawn as follows:

– A probabilistic analysis model has been developed to describe the sequential cracking behavior in an HPFRC member. In the probabilistic analysis, a HPFRC member has been modeled with segments so that variations along the member has been considered for cracking strength of the concrete matrix and the maximum tensile stress attained by fibers.
– Through the comparison of the experimental and analytical results, the tensile behavior of an HPFRC member was significantly overestimated when a single segment model was considered in the analysis. This indicates that the fiber tension model designated for a conventional FRC member should not be directly applied for an HPFRC member exhibiting multiple cracks and strain-hardening behavior. On the other hand, when an excessive number of segments was considered, the tensile behavior of an HPFRC member was considerably underestimated.
– From the parametric study with the number of segments in the analysis, it was found that the optimal number of segments for the proposed analytical model could be obtained using an average crack spacing of 1.33 times the fiber length.
– In this paper, the proposed analysis has been verified through a comparison with the test result of the HPFRC members with end-hooked steel fibers. Since the verification was conducted with only one class of test results, more verification studies are encouraged. In addition, further studies are required to investigate the tensile behavior of HPFRC members with other types of fibers such as straight steel fibers, polypropylene fibers, etc.
– The proposed analytical procedure can be employed to develop a constitutive model such as stress-strain response of HPFRC, which can be implanted on a nonlinear analysis for HPFRC members or structures. Further study is required with this research topic.

## Figures and Tables

**Table 1:** Predictions on the tensile stresses and corresponding sum of crack widths.

Index	Position in Figure 2	N_seg_				
		1	3	5	10	20
Tensile stress (MPa)	A	3.395 (0.000)	3.387 (0.081)	3.382 (0.076)	3.380 (0.069)	3.376 (0.064)
	B	-	3.683 (0.071)	3.629 (0.062)	3.585 (0.057)	3.557 (0.051)
	C	-	3.963 (0.074)	4.140 (0.073)	4.215 (0.082)	4.156 (0.078)
	D	6.022 (0.000)	5.045 (0.162)	4.714 (0.141)	4.378 (0.109)	4.191 (0.081)
Sum of crack widths (mm)	A	0.000 (0.000)	0.000 (0.000)	0.000 (0.000)	0.000 (0.000)	0.000 (0.000)
	B	-	0.030 (3.360)	0.025 (3.869)	0.025 (3.804)	0.020 (4.167)
	C	-	0.049 (1.878)	0.131 (1.204)	0.312 (0.841)	0.445 (0.885)
	D	0.574 (0.000)	0.747 (0.254)	0.811 (0.338)	0.829 (0.440)	0.797 (0.471)

Note: The numbers in parentheses are coefficients of variation (CoVs).
